# Social and health policies or interventions to tackle health inequalities in European cities: a scoping review

**DOI:** 10.1186/1471-2458-14-198

**Published:** 2014-02-24

**Authors:** Mariona Pons-Vigués, Èlia Diez, Joana Morrison, Sergio Salas-Nicás, Rasmus Hoffmann, Bo Burstrom, Jitse P van Dijk, Carme Borrell

**Affiliations:** 1 Agència de Salut Pública de Barcelona, Barcelona, Spain; 2 CIBER Epidemiología y Salud Pública (CIBERESP), Madrid, Spain; 3 Institut Universitari d’Investigació en Atenció Primària Jordi Gol (IDIAP Jordi Gol), Barcelona, Spain; 4 Institut d’Investigació Biomèdica (IIB Sant Pau), Barcelona, Spain; 5 Department of Epidemiology and Public Health, University College London, London, UK; 6 Erasmus Medical Center, Rotterdam, Netherlands; 7 Department of Public Health Sciences, Karolinska Institutet, Stockholm, Sweden; 8 Department of Community & Occupational Health, University Medical Center Groningen, Groningen, Netherlands; 9 Graduate School Kosice Institute for Society and Health, Safarik University, Kosice, Slovakia; 10 Universitat Pompeu Fabra, Barcelona, Spain

**Keywords:** Health inequalities, Public policies, Interventions, Cities, Urban health, Scoping review

## Abstract

**Background:**

Health inequalities can be tackled with appropriate health and social policies, involving all community groups and governments, from local to global. The objective of this study was to carry out a scoping review on social and health policies or interventions to tackle health inequalities in European cities published in scientific journals.

**Methods:**

Scoping review. The search was done in “PubMed” and the “Sociological Abstracts” database and was limited to articles published between 1995 and 2011. The inclusion criteria were: interventions had to take place in European cities and they had to state the reduction of health inequalities among their objectives.

**Results:**

A total of 54 papers were included, of which 35.2% used an experimental design, and 74.1% were carried out in the United Kingdom. The whole city was the setting in 27.8% of them and 44.4% were based on promoting healthy behaviours. Adults and children were the most frequent target population and half of the interventions had a universal approach and the other half a selective one. Half of the interventions were evaluated and showed positive results.

**Conclusions:**

Although health behaviours are not the main determinants of health inequalities, the majority of the selected documents were based on evaluations of interventions focusing on them.

## Background

Disadvantaged populations have worse health and higher morbidity and mortality [1]. These inequalities are due to different opportunities and resources in relation to health according to social class, gender, country of origin, territory and age [2,3]. Health inequalities tend to be greater in urban areas with disadvantaged and poor populations, affecting, as a result, all city residents [4]. Although cities offer many opportunities, jobs and services, their density, diversity, urban segregation and heterogeneous socio-economic characteristics contribute to inequalities in health [5].

**Figure 1:**
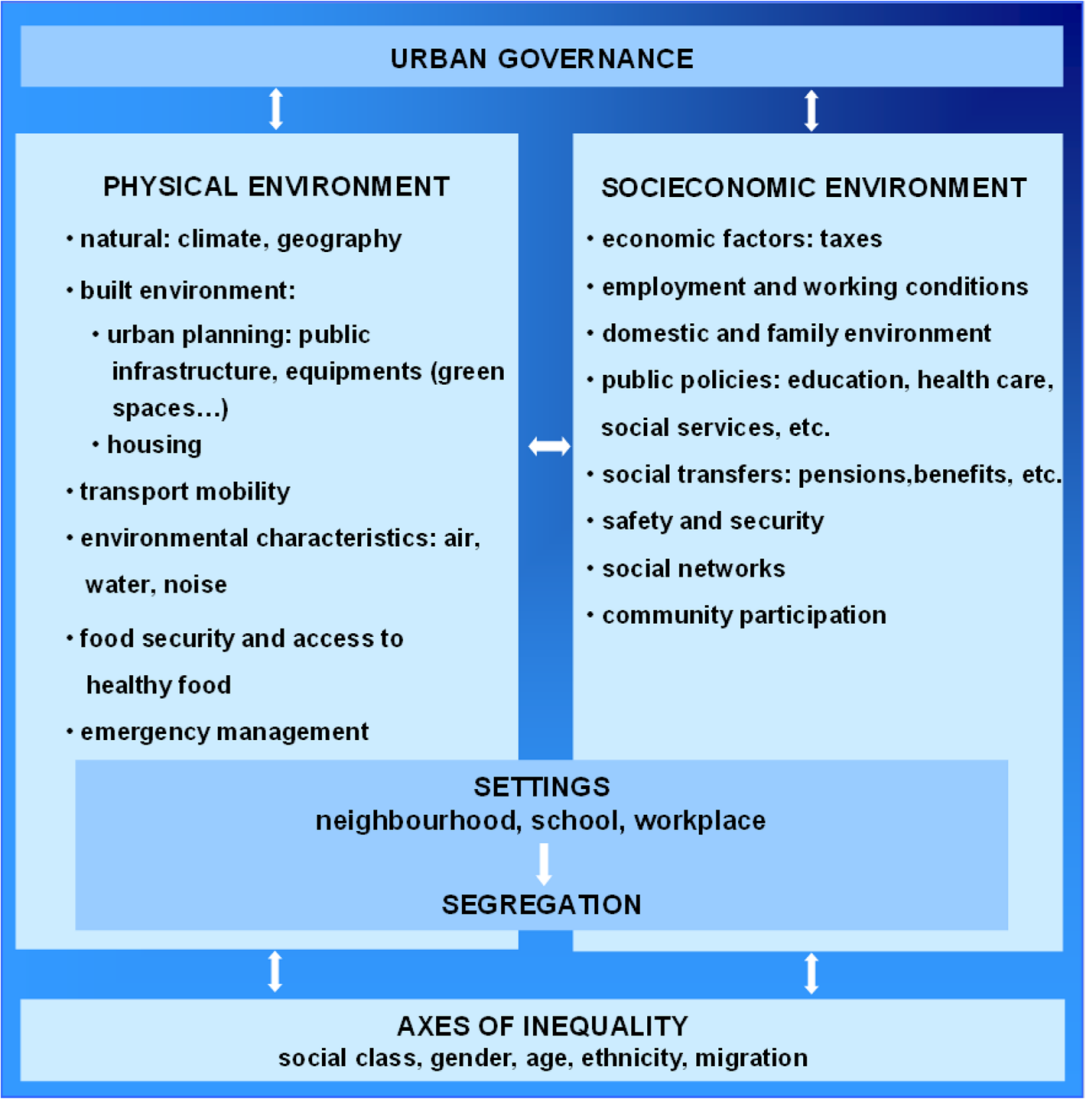
**The conceptual framework for the social determinants of health inequalities in cities of Europe [**[8]**].**

Research on the association between place or environment and health and well-being has been carried out by many scholars from a diversity of disciplines focusing on assessing differences between and within cities [6]. This research has permitted identification of the determinants of health inequalities in urban areas or neighbourhoods which are: the physical context (e.g. environmental characteristics, etc.) including the built-up environment (e.g. urbanisation and housing) and the socioeconomic context (e.g. economic factors, employment, public services, safety, etc.) (see Figure 1). Furthermore, the way in which cities are organised and managed has an impact on health inequalities, and can contribute to exacerbate or reduce them [7-9]. Health in urban populations is in turn shaped by municipal determinants and global and national trends [6]. Cities are complex systems, and, in consequence, urban health depends on many interactions [7].

Health inequalities can be tackled with appropriate health and social policies cutting across sectors, involving all community groups and governments, from local to global [4]. Governance has been defined as the relevant process by which governments and other social organizations and actors interact with citizens and make decisions in a complex and globalized world [10]. Effective multilevel governance is clearly cross-sectoral and participative. Despite the diversity of countries’ regulations, local governments have the power to face health inequalities. Municipal sectors such as urban planning, culture, leisure, education, environment, health services, social services, housing, etc. have a clear impact on the health of the citizens [7-9].

Socioeconomic inequalities in health have been recognized at least since the 1980s as being an important public health issue [11]. Europe was the only World Health Organization (WHO) Region to develop its own targets for the Health For All (HFA) strategy in 1985, with Target 1 of the 38 Targets focusing on the reduction of inequalities in health [9,12].

However, only 1.7% of the documents published in the PubMed database related to Epidemiology or Public Health mention inequalities or determinants of health in the title or abstract [13]. The majority of these health inequalities studies have been conducted to describe and analyse inequalities and are not focused on policies or interventions to reduce them in urban areas [14-17].

Although some countries are increasingly working to achieve health equity, there is little literature describing effective experiences and even less at the urban level. Given that the majority of the world population lives in cities, where health inequities are increasing [4] and that local governments have widely ranging capacities to intervene upon them, an updated review on published interventions to reduce health inequalities in cities may be helpful for future related actions. Therefore, the objective of this study was to carry out a scoping review on social and health policies or interventions to tackle health inequalities in European cities published in scientific journals. The potential research questions revolve around what is published, how this evolves, and what types of studies predominate in European cities. For this reason, evaluating the effectiveness of interventions was not the principal aim of this scoping review.

This study forms a part of the Ineq-Cities project: Socioeconomic inequalities in mortality – evidence and policies in cities of Europe (funded by DG-SANCO of the European Union); for that reason, the review focused on European cities. Hence, the project aims to study socioeconomic inequalities in mortality within European cities, as well to identify social and health policies and interventions implemented to address them.

## Methods

### Data sources

A scoping review [18,19] was carried out to select papers on policies or interventions to tackle inequalities in health in European urban areas published in scientific journals. We sought primarily to perform a mapping of the published papers; therefore, the main objective was not to assess the quality. Consequently, we opted for a scoping review which has been described as a process of mapping the existing literature or evidence base, exploring it without describing findings in detail [18,19]. In this complex topic, a scoping review is an important and necessary first step before undertaking a more intensive knowledge synthesis because it helps identify appropriate parameters and potential scope [19].

A bibliographic search was done in the National Library of Medicine’s PubMed database and in the CSA Sociological Abstracts database. Biomedical and social databases were chosen to cover different fields and disciplines related to the topic. In addition, a manual search in the reference lists of the papers selected through these two electronic databases was performed [20]. These sources were combined in order to capture as much relevant information as possible. Table 1 illustrates the search strategies and the inclusion and exclusion criteria. The search was limited to papers related to European cities and published between January 1995 and March 2011. Additional requirements for papers were: 1) presentation of the execution and/or results directly related to interventions or policies to reduce health inequalities (interventions had to state the reduction of health inequalities among their objectives), 2) written in English, Spanish or French, and 3) published in peer-reviewed journals. Theoretical papers and those only mentioning health inequalities as a conclusion or recommendation were excluded. As the research questions were what is published, how this evolves and what types of studies predominate, papers were not excluded due to the methodology used.

**Table 1:** Search strategy applied in Pubmed and Sociological Abstracts databases

**INCLUSION criteria**	European Cities
	Papers published between January 1995-March 2011
	Papers published in peer-reviewed journals
	Papers on policies or interventions to reduce health inequalities
	Papers in English, Spanish or French
**EXCLUSION criteria**	Theoretical papers on policies or interventions to reduce health inequalities
	Papers that only talk about reducing inequalities in the conclusions or recommendations
**Pubmed search**	(health equit* [Title/Abstract] OR health inequalit* [Title/Abstract] OR health inequit* [Title/Abstract] OR disparit* [Title/Abstract] OR depriv* [Title/Abstract] OR social justice* [Mesh]) AND (policy [Mesh] OR policy making [Mesh] OR health policy [Mesh] OR organizational policy [Mesh] OR health plan implementation [Mesh] OR community health planning [Mesh] OR government program [Mesh] OR program evaluation [Mesh] OR intervention* [Title/Abstract] OR policy* [Title/Abstract] OR policies [Title/Abstract] OR plan [Title/Abstract] OR action [Title/Abstract]) AND (Urban renewal [Mesh] OR Urban Health [Mesh] OR urban regeneration [Title/Abstract] OR Cities [Mesh] OR city planning [Mesh] OR urban area [Title/Abstract] OR city* [Title/Abstract] OR cities [Title/Abstract] OR healthy city* [Title/Abstract] OR municipal* [Title/Abstract] OR town [Title/Abstract])
**CSA Sociological abstracts search**	((TI = ((health inequit*) or (health inequalit*) or (health inequit*)) or TI = (disparit* or depriv*)) or (AB = ((health inequit*) or (health inequalit*) or (health inequit*)) or AB = (disparit* or depriv*)) or (DE = (social justice*))) and ((DE = (policy or (policy making) or (health policy)) or DE = ((organizational policy) or (health plan implementation) or (community health planning)) or DE = ((government program) or (program evaluation))) or (TI = (intervention* or policy* or policies) or TI = plan) or (AB = (intervention* or policy* or policies) or AB = plan)) and ((DE = ((urban renewal) or (urban health) or cities) or DE = (city planning)) or (TI = ((urban area) or city* or cities) or TI = ((health city*) or municipal* or town)) or (AB = ((urban area) or city* or cities) or AB = ((health city*) or municipal* or town)))

### Study selection

**Figure 2:**
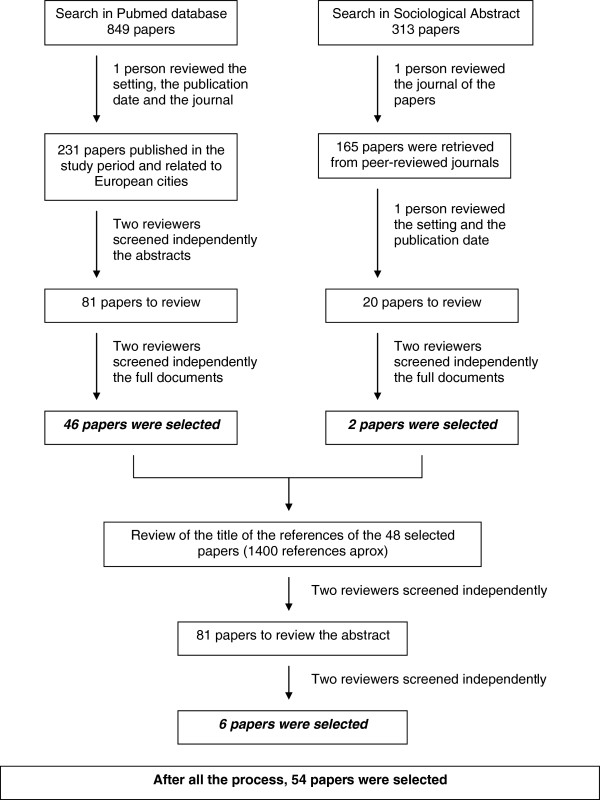
Literature review and data abstraction flow chart.

Figure 2 represents the data extraction flow chart. The searches in Pubmed and Sociological Abstracts were carried out on the 1st of April 2011, and identified 1162 papers (849 papers from Pubmed and 313 from Sociological Abstracts). All these citations were systematically screened and evaluated by one author to exclude publications irrelevant to the inclusion criteria. Therefore, according the Pubmed search, 231 papers related to European cities had been published in the study period. Two reviewers independently screened these 231 abstracts, and 81 papers were selected to be completely reviewed. Finally, 46 of these papers were included in the study.

With reference to the Sociological Abstracts search, 165 papers were retrieved from peer-reviewed journals. Nevertheless, only 20 of them were related to European cities and published in the study period. Two reviewers independently screened these 20 papers and two of them were included in the study. In both searches, the disagreement between the reviewers about 22 documents was resolved by a third reviewer.

The titles of the documents referenced in the 48 papers selected were screened to exclude obviously irrelevant or duplicate documents. After that, their abstracts were screened independently by two reviewers (MP, JM) who had previously screened the papers in order to find additional ones to be included. The same criteria formerly used were applied. This manual search in the selected papers’ reference lists led to the identification of 6 new papers. In consequence, the entire review process resulted in the selection of 54 papers.

### Data extraction, variables and data analysis

The following data were extracted from each publication: authors, year of publication, goal of the paper, study design, city and year of the intervention, target population of the intervention, intervention description, evaluation of the intervention and results or health outcomes of the paper. This information for each paper was captured in a table displayed in the annex of this review (see Additional file 1: Tables S1-S5), and in a database. Five researchers participated independently in the study selection and the data extraction.

The main characteristics of the documents were classified and are summarized in Tables 2 and 3, taking into account the following variables:

– Type of intervention to reduce health inequalities: 1) Promotion of health behaviours, 2) Healthy settings (where people actively use and shape the environment; as for example, school, workplace, neighbourhood and community), 3) Socioeconomic context (economic factors, employment and working conditions, public services and social transfers), 4) Physical context (climate, built-up environment and environmental characteristics) and 5) Combined approach. This taxonomy was based on the IneqCities project conceptual framework [8,21], (Figure 1), adapted from the one proposed by the WHO Hidden Cities Report [4] and from Vlahov [5].

– Study design of the paper: documents were classified according to the design type of the research conducted. The categories were: experimental studies, quasi-experimental studies, observational studies, qualitative studies, reviews, health impact assessment studies and mixed-methods (studies combining two or more designs).

– Country of city intervention: this variable described the cities where interventions were developed according to the home country. An extra category was added when the intervention took place simultaneously in different cities in different European countries.

– Setting of the intervention: this variable indicates where the intervention was performed and it was categorised as: the entire city, a neighbourhood or area, a healthcare or social center, school, or a specific community group.

– Target population of the intervention: a variable consisting of three categories according to the age of the participants: children, adult and all ages.

– Focus of the intervention: a dichotomous variable indicating whether the intervention was universal or selective (intervention was addressed to a part of the population with some risk or vulnerable features).

– Evaluation of the intervention: this variable collects information from the paper about whether the intervention had been evaluated, and the type of result (positive, negative or partially positive-negative). Some interventions could have been evaluated but if this had not been indicated in the paper it was not stated. It includes different types of evaluation: efficiency, effectiveness, impact, monitoring, satisfaction, etc.

– Year of publication categorized in three groups: 1995-2000, 2001-2005 and 2006-2011.

**Table 2:** Descriptive characteristics of the 54 papers obtained classified by type of intervention

	**Type of intervention**					**Total**	
	**Promote healthy behaviours**	**Promote healthy settings**	**Socio-economic context**	**Physical context**	**Combined approach**	**N**	**%**
	**(N = 24 - 44.5%)**	**(N = 10 – 18.5%)**	**(N = 6 – 11.1%)**	**(N = 4 – 7.4%)**	**(N = 10 – 18.5%)**		
**Study design**							
Experimental	11	3	2	0	3	19	35.2
Quasi-experimental	5	5	0	1	2	13	24.1
Observational	5	1	2	1	0	9	16.7
Qualitative	1	1	0	0	3	5	9.2
Review	1	0	0	2	2	5	9.2
Health impact assessment	0	0	2	0	0	2	3.7
Mixed methods	1	0	0	0	0	1	1.9
**Country**							
United Kingdom	19	9	4	4	4	40	74.1
Spain	0	0	2	0	3	5	9.2
Netherlands	4	0	0	0	0	4	7.4
Germany	0	0	0	0	2	2	3.7
France	1	0	0	0	0	1	1.9
Different European countries	0	1	0	0	1	2	3.7
**Setting**							
City	3	2	4	3	3	15	27.8
Neighbourhood	6	3	1	1	2	13	24.1
Healthcare center	11	0	0	0	1	12	22.2
School	2	4	1	0	2	9	16.7
Community group	2	1	0	0	2	5	9.2
**Target population**							
Adults	14	1	1	0	2	18	33.3
Children	4	6	0	1	2	13	24.1
Adults and Children	6	3	5	3	6	23	42.6
**Focus**							
Universal	10	6	3	3	5	27	50.0
Selective	14	4	3	1	5	27	50.0
**Evaluation of the intervention**							
Yes, positive result	13	6	3	1	6	29	53.7
Yes, negative result	5	1	0	0	2	8	14.8
Yes, positive and negative results	2	1	1	3	0	7	13.0
Yes, missing result	0	2	0	0	0	2	3.7
No	1	0	1	0	0	2	3.7
Missing	3	0	1	0	2	6	11.1
**Year of publication**							
2006**–**2011	14	6	2	0	4	26	48.2
2001**–**2005	7	1	4	4	4	20	37.0
1995-2000	3	3	0	0	2	8	14.8

**Table 3:** Interventions classified according to type of intervention, setting and target population

**Type of intervention**	**Setting**	**Target population**		
		**Adult**	**Children**	**Adult & Children**
**Promote healthy behaviours**	**City**	Physical and cognitive training programme		Health check tests and a poster campaign
		Different activities to promote healthy eating		
	**Neighbourhood or area**	A weight management programme	Reduce teenage pregnancy	Introduction of health brokers to promote health
		Physical exercise program	Dietary health education program	
				Intensive home-visiting program
	**Healthcare or social center**	A web-based intervention to support self-management in patients with coronary heart disease	Oral health promotion program	Interventions to prevent mother-to-child transmission of HIV
		Assessing CVD risk in pharmacies		Introduction of asthma specialist nurses
		Preventive care of patients with high CV risk		
				Individual asthma education programme
		Cultural approach to CV risk prevention program		
		Behavioural counselling on fruit and vegetables consumption		
		Health shop in community centre		
		Cognitive Behaviour Theraphy for smokers		
	**School**		Teacher-supervised toothbrushing	Oral health promotion program
	**Community group**	Intervention for diabetes support and education		
		Health education and physical exercise programme		
**Promote healthy settings**	**City**		Domestic water fluoridation	
	**Neighbourhood or area**		Receiving pasteurised cows’ milk by 6 months of age	Free smoke alarms and fire safety information
				Improved physical access to high-quality ‘healthy’ foods
				Installation of central heating
	**Healthcare or social center**			
	**School**		Road safety program	
			Free healthy school meals	
			Playground redesign intervention	
	**Community group**	Impact of English smoke-free legislation		
**Socioeconomic context**	**City**	Food services and insertion for the homeless		Primary care reform
				Transport strategy
	**Neighbourhood or area**			Early Years Centers provide high quality day care
	**Healthcare or social center**			
	**School**			Providing daycare facilities for young children
	**Community group**			
**Physical context**	**City**		Traffic calming	Urban regeneration programs
				Interventions to improve housing
	**Neighbourhood or area**			Neighbourhood renewal
	**Healthcare or social center**			
	**School**			
	**Community group**			
**Combined approach**	**City**			Healthy cities
				Strategies to reduce health inequalities
	**Neighbourhood or area**			Family Support Services
				Social and health maternal and child intervention
	**Healthcare or social center**			Postnatal Support Health Visitor
	**School**		Environmental and educational intervention	
	**Community group**	Race equality policies		
		Social care and health follow-up programme targeting homeless tuberculosis patients		

CVD: Cardiovascular diseases.

CV: Cardiovascular.

HIV: Human Immunodeficiency Virus.

## Results

The entire review process led to the selection of 54 papers. The main characteristics are presented in Table 2 and 3 summarizes the objective according to the type of intervention, the setting and the target population.

Although publication of interventions tackling health inequalities has increased considerably over the last 15 years, the number of documents is still very small. Between 1995 and 2000, six papers were published, whereas in the past 6 years (2006 - April 2011), 26 papers had been published (48.2% of the documents of this review). Nineteen of the papers reviewed (35.2%) used an experimental design such as randomised controlled trials, followed by 13 quasi-experimental studies (24.1%). 16.7% of the documents (9 papers) were observational studies whereas 9.2% were review (5 papers) and qualitative studies (5 papers).

Forty of the 54 studies (74.1%) were carried out in cities of the United Kingdom. The 18 remaining papers were divided between cities in Spain (5 studies, 9.2%), the Netherlands (4 studies, 7.4%), Germany (2 studies, 3.7%), France (1 study, 1.9%) and other European countries (2 multicentric studies, 3.7%). The setting of the intervention is more evenly distributed. The setting of 15 papers was the whole city (27.8%), in 13 it was a deprived neighbourhood (24.1%) and in 12 a healthcare or social center (22.2%).

The majority of the interventions targeted both adult and child populations (23 studies, 42.6%). Half of them used a universal approach, and the other half a selective one. The majority of the interventions were evaluated (46 interventions, 79.3%) and 29 of them had positive results (53.7%). The rest of the evaluation showed that 8 interventions (14.8%) were not effective, 7 (13.0%) showed both positive results and negative results, and 2 interventions (3.7%) did not mention the results of their evaluation. Not all of the evaluated interventions used health indicators as outcome measures. Further details of the evaluation of each intervention or policy are available in the supplementary data.

Almost half of the interventions promoted healthy behaviours (24 documents, 44.5%), for example to promote oral health programmes, increase physical activity, decrease cardiovascular risk factors, etc.; 10 interventions (18.5%) promoted healthy settings, for example to improve road safety, redesign a playground, etc.; 6 interventions (11.1%) targeted the socioeconomic context, for example: transport policy, food services, etc.; 4 interventions were focused on the physical context (7.4%), for example: neighbourhood renewal, etc.; and 10 interventions (18.5%) used a combined approach, for example: environmental and educational interventions to promote water consumption. Further details of the documents employing these five approaches are available in the supplementary data.

More than half of the interventions promoting health behaviour had adults as the target population (14 papers), 14 papers used a selective approach, 13 papers were evaluated and found positive results. Eleven of these 24 interventions were set in healthcare centers. Examples of interventions developed in health centres and aimed at the adult population are behavioural counselling on fruit and vegetables consumption [22], and setting up a health shop in a community centre [23].

Six of the 10 interventions promoting healthy settings targeted children, had a universal approach, were evaluated and showed positive results. Four of these interventions were developed at schools, for example, playground redesign interventions [24,25].

Five of the 6 papers about interventions related to socioeconomic context and material resources were addressed to the whole population, three papers took a universal approach, four papers addressed the whole city and 4 papers were evaluated and found positive results. Primary health care reform [26] was an example of intervention aimed at the entire population of the city.

The physical context was the focus of only 4 interventions. Three of them targeted the whole population, were evaluated and obtained both positive and negative results.

More than half of the interventions using a combination approach targeted the whole population (6 papers), used a universal approach (5 papers), were evaluated and obtained positive results (6 papers). Three of these interventions addressed the whole city, for example interventions related to the healthy cities strategy [27].

During the review process some protocols of design, implementation or interventions evaluation were identified. Although they were out of the scope of this study, we considered that they provided interesting information. For that reason, these 4 studies are cited in this paper [28-31].

## Discussion

This project identified 54 papers published in scientific journals related to social and health policies or interventions to tackle health inequalities in European cities. A third of the studies used an experimental design, most of them were carried out in the United Kingdom, a quarter had either the whole city as the setting or a deprived neighbourhood and half of them promoted healthy behaviours. The majority of the interventions targeted both adults and children, half of them used a universal approach and the other half a selective one. Half of the interventions were evaluated and had positive results.

Although the volume of publications in this field has increased over the years, there are still relatively few papers published in scientific journals related to policies and interventions focussed on real experiences to tackle health inequalities. Different factors may contribute to this fact. As the literature shows, searching for studies on the social determinants of health or health inequalities is difficult and time-consuming [16]. Although the combination of heterogeneous sources of data were included in order to include as much relevant information as possible, there are probably many documents describing interventions to reduce social inequalities in health which have not been collected in this review because they are in the grey literature (for example, European Portal for Action on Health Inequalities [32]) or because there is no evidence of this purpose in the objective or in the summary of the paper. This has already been identified as a real gap in a review paper on the wider social determinants of health [16]. As we had a more specific aim, it is probable that there is even less available scientific literature. Although some useful interventions have been published in grey literature and reports or in presentations [12], it would be interesting to publish them in scientific journals as this is the best way to reach scholars and improve evidence based scientific knowledge.

In addition, we were looking for policies and interventions at the local level. The reduction of health inequalities cannot be done only with policies undertaken in urban areas; there are many health determinants which are the responsibility of the national or regional government [3,15]. Some important universal policies with great potential effect on health inequalities, implemented on a national level, may therefore be overlooked (e.g. maternal and child health services). However, local governments have certain competences in a number of sectors from which they can contribute in the reduction of socio-economic health inequalities [33]. As cities are an important setting for intervention governance, it is necessary to show what is being done at this level. In the health policy documents of some cities or counties, it is possible to see that certain strategies are planned [34], but often these strategies do not end up being described to the scientific community. This may be due to the heavy workload of people responsible for such interventions, but also because it is very difficult to publish them as local experiences are not as attractive to scientific journals as national or international strategies. As social health inequalities are an important public health issue, it might be interesting to promote publishing of strategies carried out, or at least to increase the number of published interventions in scientific journals. Health inequalities are present in all countries; therefore, the experience of interventions carried out in other cities to tackle them might prove to be very interesting and helpful.

In children and in adults, interventions are mainly focussed on the lifestyle drift and on promoting healthy behaviours [35]. As Bambra [36] reported, policy has had a tendency to focus on healthy behaviour interventions rather than tackling upstream, distal causes such as poor living conditions. This approach contrasts with evidence which suggests that health inequalities tend to persist between socioeconomic groups even if lifestyle factors are equalized [36]. This poor progress is probably attributable to the fact that studying the impact on health inequalities of interventions in upstream structural determinants is more difficult due to the complex pathways between these determinants and health inequalities. However, the recent WHO review of social determinants suggests that addressing the “causes of the causes” is the right way to proceed on these, ensuring that people have the skills and control over their lives to be able to change behaviour [37].

Although many cities are making efforts to tackle inequalities in health [12], it is necessary to move from a traditional health education focus on lifestyle determinants to more strategic interventions on structural determinants (as many of those presented in Figure 1). The challenge of reducing health inequalities requires policies and interventions in all sectors of society, because their causes are complex [6,7,9]. That is to say that health inequities challenge the traditional division of societies and their governments into sectors for organizational purposes [35]. Many policies aiming to address social determinants require intersectoral action [38] and community participation, two principles of action proposed by the Commission on Social Determinants of Health [15]. This is especially relevant in cities, according to statements by Healthy Cities [39]. A recent paper suggests that intersectoral approaches to health equity are feasible in a variety of social, economic and political systems [38]. In consequence, the intersectoral approach is essential to improve the health, wellbeing and quality of life of citizens. All community sectors are essential in addressing health policy [40]. Unfortunately, these aspects are not too clear or evident in many of papers in this review. Achieving community participation and working intersectorally is not an easy task, even though it is considered important [40]. Evidence shows that intersectoral action is more effective than tackling health inequalities from the health sector only [41]. A useful tool to promote effective work in this sense is the Urban Health Equity Assesment and Response Tool (Urban Heart), a clear guide for policy makers at local and national levels to address health inequalities in cities [42]. Moreover, intersectoral action facilitates the WHO policy Health in All Policies, which takes into account the impact on health of all policies, such as education, environment, economic policies, housing, or transport mobility [43]. In addition, current experience shows that health impact assessment could play an important role in the development of the Health in All Policies strategy. Health impact assessment is an important methodology to support decisions in policy-making [44].

The United Kingdom (UK) is one of the countries with a longer historical background of working on health inequalities [45,46] and, for example, London has a specific strategy to tackle health inequalities [34] which has been supported by different governments. This fact is corroborated in this paper because the UK is the country where most published interventions were found. In addition, English people might have more facilities to publish because of the language. We found more papers in Pubmed than in the Sociological Abstracts database probably because the latter tends to focus on international literature in sociology and disciplines traditionally more related to the social and behavioural sciences rather than to health. However, social and economic factors are being considered more and more as determinants of health inequalities so we consider that this trend may change. In consequence, this issue may deserve further research.

There is still little experience of evaluating the impact of interventions to reduce health inequalities [27,47,48]. Most of the evaluations reviewed in this study concerned healthy behaviours and proximal outcomes. Evaluating upstream interventions presents additional methodological challenges. Evidence is especially needed from evaluations of universal polices [35]. That is particularly important to inform the debate on the most efficient and effective use of scarce resources in financial crisis situations, such as the present global one. In addition, it is necessary because it can happen that some interventions may increase inequalities in the population if they are of greater benefit to advantaged (lower-risk) groups than to disadvantaged (higher-risk) groups [49]. Despite the fact that the aim of this scoping review was not to evaluate the effectiveness or quality of the interventions included [18,19], this is a subject which warrants further study. Note that 54% of the interventions were evaluated and had positive results. However, not all reported results in terms of health indicators. Even in the UK which has such a long history of tackling social determinants of health, evaluation is sorely lacking [50]. About 35% of the papers used an experimental design to evaluate the intervention. Overall, the evaluation of effects is the aspect most commonly published in scientific journals rather than accounts of real experiences, which are probably explained to other audiences.

### Strengths and limitations of the study

To our knowledge, this is the first scientific review of the social and health policies or interventions to tackle health inequalities in European cities. Previous papers have reviewed health inequalities interventions at the national level or at the urban level but in the American context (USA and Canada) [33,36]. For that reason, this study can contribute to advancing knowledge in the reduction of socioeconomic health inequalities. At the national level, Bambra et al. combined a rapid review with a Delphi distillation, producing a shortlist of evidence-based recommendations. According to these authors, extensive, specific and robust evidence is urgently needed to guide policy and programmes [36]. Besides, Collins and Hayes demonstrated a pervasiveness of ‘behavioural’ and ‘biomedical’ perspectives, and a lack of consideration afforded to the roles and responsibilities of municipal governments, among the health inequities scholarly community [33].

The rigorous procedures used have ensured the validity of the data abstraction. However, the review is not exhaustive because we have not been able to identify all relevant evidence and we did not search in the grey literature. We tried to identify all possible papers published in scientific journals by searching in two different databases, one of which is not medical, in order to capture policies or interventions from other sectors. The combination of heterogeneous sources of data adds value to the results. As one of the additional requirements for inclusion was that papers were written in English, Spanish or French, we believe it has brought a significant change in the results since we only had to exclude two documents.

We must also take into account that the fact that these policies and interventions have been published does not necessarily mean they are still functioning or effective. Furthermore, given the heterogeneity of the objectives of the papers it has not been possible to summarize their main results or conclusions. The articles varied with regard to the richness of information included to describe different aspects of these interventions or policies.

## Conclusions

The volume of publications in this field has increased over the years, however, there are still relatively few papers published in scientific journals related to policies and interventions focussed on real experiences to tackle health inequalities. In addition, although health behaviours are not the main determinants of health inequalities [15], the majority of the documents published in scientific journals were based on interventions focusing on them. In order to increase the policy evidence, to support people working in interventions and to contribute to the incorporation of health in all policies [15], examples of local policies and interventions to reduce inequalities should be published in the scientific literature.

## Competing interests

The authors declare that they have no competing interests.

## Authors’ contributions

MPV, ED, JM and CB are the principal investigators responsible for the conception of the project. In addition, MPV, ED, JM and CB were involved in data collection, analysis of the data and writing the manuscript. SSN, RH, BB and JPD have made substantial contributions to interpretation of data; have been involved in drafting the manuscript or revising it critically for important intellectual content. All authors participated in the discussion of the analysis, the editing of the drafts and read and agreed on the final version of this manuscript. All authors have participated sufficiently in the work to take public responsibility for appropriate portions of the content. All authors read and approved the final manuscript.

## Pre-publication history

The pre-publication history for this paper can be accessed here:

http://www.biomedcentral.com/1471-2458/14/198/prepub

## Supplementary Material

Additional file 1: Table S1 Promotion health behaviours interventions. **Table S2.** Healthy settings interventions. **Table S3.** Socioeconomic context interventions. **Table S4.** Physical context interventions. **Table S5.** Combined approach interventions. List of the 54 papers selected in this study. Click here for file
