# Supplementary material for: Social and health policies or interventions to tackle health inequalities in European cities: a scoping review
Source: BMC Public Health. 2014 Feb 24;14:198. doi: 10.1186/1471-2458-14-198 (PMC3938820; doi:10.1186/1471-2458-14-198)
Supplement: Additional file 1: Table S1 — Promotion health behaviours interventions. Table S2. Healthy settings interventions. Table S3. Socioeconomic context interventions. Table S4. Physical context interventions. Table S5. Combined approach interventions. List of the 54 papers selected in this study. [file 1471-2458-14-198-S1.doc]

## Additional file 1

## Table S1: PROMOTION HEALTH BEHAVIOURS INTERVENTIONS

| **AUTHOR(S)/**  **YEAR** | **GOAL PAPER** | **STUDY DESIGN OF THE PAPER** | **CITY**  **(COUNTRY) and YEAR** | **TARGET POPULATION OF THE INTERVENTION** | **ACTION/**  **INTERVENTION** | **EVALUATION OF THE INTERVENTION** | **RESULTS / HEALTH OUTCOMES** |
| --- | --- | --- | --- | --- | --- | --- | --- |
| **ID 25**  Kerr et al., 2010 | To explore the effectiveness of a web-based intervention in decreasing inequalities in access to self-management  support in patients with coronary heart disease (CHD) | Quantitative and qualitative methods.  Prospective cohort study on the level of use of a web-based intervention by primary care patients with CHD over a 9-month period.  Qualitative methods | North London (UK) | Patients with a diagnosis of CHD from primary care centers in diverse socioeconomic and ethnic areas of North London | Comprehensive Health Enhancement and Social Support (CHESS) Living with Heart Disease web-based intervention  used in this study provided interactive information, behavior change support, and peer and expert support components | This article performs the evaluation | Only 10.6% of eligible patients participated. Most Caucasian well-educated young men. Greater use of the intervention in older and in those that had previously home Internet access (OR 3.74). The availability of a web-based intervention, with support for use at home or through public Internet services, did not result in a large number or all types of patients with CHD using the intervention for self-management support. |
| **ID 31**  Harting et al., 2010 | To explore the possibilities of institutionalizing a comparable role for a ‘health broker’ in four Dutch municipalities as an additional investment to promote health in deprived neighbourhoods | Cross-sectional assessing the interest and utility of the intervention | Four municipalities  (Netherlands)  Years 2007-2008 | Population of the neighbourhoods chosen (mainly poor) | Introduction of health brokers to promote health in the district | Qualitative assessment of the role of the health-brokers | Health brokers can create additional opportunities to gradually strengthen local health promotion efforts |
| **ID 135**  Hajek et al., 2010 | Two pilot studies were undertaken to assess the efficacy and potential reach of a weight management programme in two inner-city areas of East London | Piloting the intervention | London (UK)  Years 2005 and 2007 | Residents in inner-city London | Participants were treated in groups of 7–18 people. The programme comprised 6 weekly treatment sessions and two follow-up visits lasting 1 h each. The treatment goal was to lose one pound (0.45 kg) a week | They analyse the results (before and after) of the programme without control group | A task-based lifestyle-modification programme complemented by social support proved attractive to inner-city residents and generated significant short-term effects comparable to those achieved by intensive interventions with more traditional target groups |
| **ID 148**  Horgan et al., 2009 | To investigate a targeted pharmacy-based CVD risk assessment service for primary prevention aiming to evaluate service feasibility, assess effectiveness  of identifying at-risk individuals and of reaching disadvantaged groups and measure referrals from the service to local general practices | Piloting the intervention | Birmingham (UK)  Years 2007-2008 | People aged 40–70 years with no CVD disease, who consented  for sharing of identifiable information with their GP  and anonymized information for evaluation | Cardiovascular risk factors, including blood pressure, nonfasting cholesterol/HDL cholesterol ratio, smoking and diabetic status, were measured by pharmacist in order to calculate a 10-year cardiovascular risk score using the Framingham equations | They present the results of the programme after the intervention | Community pharmacies can provide a CVD risk assessment service in a UK urban setting that can attract males and provide access for deprived communities and Black and Asian communities. A pharmacy service can support GP practices in identifying and managing the workload of around 30% of clients |
| **ID 166**  Schuring et al., 2009 | To evaluate the effectiveness of a health promotion programme,  consisting of physical exercise and cognitive training, on the physical and mental health of unemployed subjects with health complaints | Randomised controlled trial | Rotterdam (Netherlands)  Years 2004-2007 | Participants  with health problems and declared to be capable of full-time employment | Three sessions weekly over 12 weeks. One session a week was focused on education to enhance the ability to cope with  (health) problems, and 2 weekly sessions consisted of physical activities | This article performs the evaluation | This intervention programme aimed at the promotion of physical and mental health in unemployed people with health complaints did not show beneficial effects |
| **ID 252**  El Fakiri et al., 2008 | To examine the effectiveness of a structured collaboration in general practice between a practice nurse, a peer health educator, the general practitioner (GP) and a GP assistant in providing intensified preventive care for patients at high risk of developing cardiovascular diseases | A randomised controlled trial consisting of an intervention group that received intensified  preventive care and a control group that received usual GP care | Three healthcare centres in deprived  neighbourhoods of the Hague and Rotterdam (Netherlands)  Years 2002-2004 | 275 high-risk patients (30-70 years) from various ethnic groups were randomised to  intervention (n=137) or usual care group (n=138) | The intervention was based on the formation of a team consisting of a GP, a Practice Nurse (PN),  a GP assistant and a Peer Health Educator (PHE). Intervention activities were  based on a specially constructed protocol that was based on the current Dutch General  Practice Guidelines for hypertension, hypercholesterolemia, diabetes and smoking | This article performs the evaluation | The cardiovascular risk profile of intervention and control patients improved after one year follow-up.  The present study shows no benefits of adding a PN and a PHE to the general practice on cardiovascular risk among high-risk patients living in deprived neighbourhoods |
| **ID 272**  Bellary et al., 2008 | To investigate the effectiveness of a culturally sensitive, enhanced care package in UK general practices for improvement of cardiovascular risk factors in patients of south Asian origin with type 2 diabetes | A cluster randomised controlled trial, 21 inner-city practices in the UK were assigned by simple  randomisation to intervention  or control | Coventry and Birmingham (UK)  Years 2004-2007 | All adult patients of south Asian origin with type 2 diabetes | Enhanced care including additional time with practice nurse and support from a link worker and diabetes-specialist nurse | This article performs the evaluation | Significant differences were recorded between treatment groups in diastolic blood pressure and mean arterial pressure. No differences were found in other measures (cholesterol, systolic blood pressure) |
| **ID 273**  Tubert-Jeannin et al., 2008 | To evaluate an oral health promotion programme being carried out with children at high risk for cavities located in deprived neighbourhoods | Randomised control trial of schools. Two schools with full intervention, 2 with incomplete intervention and 5 control schools | Clermont-Ferrand (France).  Years 2005-2006 | Parents, teachers and children of the schools | Education and showing to promote the improvement of tooth brushing habits (2 times/day) and general oral hygiene associated with the use of fluoridated toothpastes | This article performs the evaluation | A significant improvement in children’s oral hygiene habits was obtained in the programme group as compared to the control group. However, the care and treatment needs of the children in both groups were not covered, given that one child out of four remained with untreated lesions at the end of the year |
| **ID 301**  Cochrane and Davey, 2008 | To increase physical activity in an urban community, based on the social ecology model | A quasi-experimental design was chosen to  test whether this approach can increase, significantly, the population proportion that is physically active | Burngreave and Manor, two deprived areas in Sheffield (UK) | Population of Burngreave. Emphasis was  focused on adults (≥16 years of age) poor socio-economic profiles and poor health profiles | Physical activities were  introduced in accessible community areas in five broad categories: walking, exercise referral, sports, water activities and pastimes and active leisure pursuits | This article performs the evaluation | The intervention sample demonstrated trends towards: being more physically active compared with one year ago, greater readiness to take up physical activity, better general health and improved health compared with one year ago |
| **ID 351**  Davies et al, 2007 | To measure the clinical impact of a linked series of interventions on Early Childhood Caries (ECC) and general caries levels in a community of five-year-old children | Multi-stage intervention programme was introduced to pre-school children in the non-fluoridated city of Manchester, UK | Manchester  (UK)  Years: 1997-1998 and 2003-2004 | Children of 5 years old | Health Visitors giving dental health advice and gift bags to parents when babies received their 8-month developmental checks and/or attended clinics for MMR vaccinations at 12-15 months. The ‘8-month’ bags contained trainer cups with leaflets to encourage the use of safe drinks and the ‘MMR bags’ contained toothpaste and brushes with leaflets encouraging the early commencement of twice daily supervised brushing | This article performs the evaluation | When data from all the children were analysed, regardless of whether they participated or not, the differences were no longer apparent |
| **ID 396**  McIntosh and Shute, 2006 | To gain insight into how interventions provided by health visitors in the Starting Well Project are operationalised and how they are perceived by parents | Qualitative. Semi- structured interviews at two times | Two deprived areas of a Scottish city (UK)  Years 2002-2003 | Purposive sample of 20 mothers and 9 health visitors | Intensive home-visiting schedule during the child’s first 3 years of life. Activities to engage parents in  goal-setting directed at improving their own and their infants’ health | Evaluated  (Shute & Judge, 2005) | Parental perceptions of being supported were exemplified by increased confidence in infant care, reduced anxiety regarding infant care needs, increases in knowledge and sense of personal  competence in parenting practices, reduced isolation, and advocacy for those experiencing problems |
| **ID 410**  Rae, 2006 | In this revision study two interventions are described:  **A.Camden’s Pitstop Health Check project**  To carry out health check tests to determine risk factors for a range of different health conditions.  **B. Manchester’s valuing older people positive**  **images of ageing campaign** | The results from these tests were discussed with the participants and used to design an individual health improvement programme. Each participant received a full test report covering all areas tested, with explanations of what a good or normal result  range should be together with recommendations  about any less than good results  To challenge negative stereotypes of older people | London (UK)  Year 2006  Manchester (UK)  Year 2005 | 106 people from Camden in north  London  Older population of Manchester | To perform health check tests to determine risk factors for a range of different health conditions.  A city-wide poster campaign and other media coverage was used to promote the production of the 2006 calendar and photographic exhibitions by older population | Feedback from the Pitstop Health Check participants has been very positive with 94% commending the work.  Sixty questionnaires  were dispatched and 46 were returned which  represents a 76.6% response rate | Analysis of the results showed that there were many more cases of people at risk of ill health than was initially acknowledged  Anecdotal evidence suggests that there has been an increased number of older people accessing local groups and activities. |
| **ID 413**  Mohiddin et al., 2006 | To describe the process of a local strategy to reduce teenage pregnancy in a deprived area | Needs assessment was performed and the local strategy was reviewed. Prevention and better information and understanding the local context were prioritized | East London deprived borough (UK)  Year 2004 | East London deprived teenage inhabitants | A needs assessment was performed through public health and youth services co-chairing the teenage pregnancy board. Strengthening sex and relationship education in and out of schools. Also, qualitative work was carried out with young people and men as well as reviewing sexual health services and involvement of a local charity and the media | Three separate  Government agencies scrutinized us in a 12-month period | The conception rate has fallen from 101.5 to 84 per 1000 with absolute numbers of 415 pregnancies in 2003 and 352 in 2004. Whether if this is a downward trend and whether the intervention initiated it still has to be established |
| **ID 424**  McDonald et al., 2007 | To evaluate the effectiveness of interventions to prevent mother-to-child transmission of HIV at a large teaching hospital and to assess reasons for the small numbers of transmissions that continue to occur | A database of all pregnant women diagnosed as HIV  positive between 1993 and 2005 was reviewed, with detailed retrospective case-note review of all mother–infant pairs  where HIV transmission occurred | South East London (UK)  Years 1993-2005 | 274 pregnant women from the King’s College Hospital HIV clinic | A report on the prevention of MTCT at this institution, combined with detailed case-note reviews of all mother–infant pairs where the infant was HIV infected | This article evaluates the effectiveness of the interventions to  prevent mother-to-child transmission of HIV | Low rates of mother-to-child transmission (MTCT) of HIV  are seen in this UK cohort, attesting to strong multidisciplinary  teams.  Some MTCT occurs early in utero and starting antiretroviral treatment early should be considered, particularly in women with a history of preterm labour. |
| **ID 524**  Greenhalgh et al., 2005 | To develop a complex intervention for diabetes support and education in minority ethnic groups, delivered through bilingual health advocates | Action research | Deprived inner London district (UK) | Ethnic minorities | Selection of bilingual health advocates, groups led by advocates and organisational support  Community, culture and values | This article describes the first evaluation. Later a RCT was performed  (Greenhalgh 2011) | Groups were popular and well evaluated. Action research approach allowed engagement with an underserved group. The study produced subjective benefits to these groups |
| **ID 526**  Jackson et al., 2005 | To determine whether teacher-supervised toothbrusing, once a day, at school, during term time, with flouride toothpaste could reduce dental caries in primary school children | A randomised, single-blind, parallel group clinical trial. | An area where the flouride content of the drinking water was <0.3ppm. North West London (UK)  Years 2000-2002 | 517 children in the first term of their first year of primary school (5-year-old) | Class teachers were trained individually by the same dental hygienist in an appropiate toothbrushing technique for young children. Children in the intervention group brushed once a day at school. The control group did not receive this intervention | This article performs the evaluation | This programme can be effectively targeted into socially deprived communities and a significant reduction in dental caries can thereby be achived especially among caries-susceptible children |
| **ID 580**  Griffiths et al., 2004 | To determine whether asthma specialist nurses, using a liaison model of care, reduced unscheduled care in a deprived multiethnic area | Cluster randomised controlled trial | London (UK) | 44 general practices in two boroughs in east London. 324 people aged 4-60 years admitted to or attending hospital or the general practitioner out of hours service with acute asthma | Patient review in a nurse led clinic and liaison  with general practitioners and practice nurses comprising educational outreach, promotion of guidelines for high risk asthma, and ongoing clinical support. Control practices received a visit promoting standard asthma guidelines; control patients were checked for inhaler technique | This article performs the evaluation | Asthma specialist nurses using a liaison model of care reduced unscheduled care for asthma in a deprived multiethnic health district. Ethnic groups may not benefit equally from specialist nurse intervention |
| **ID 612**  Reijneveld et al., 2003 | To assess the effect of a short health education and physical exercise programme on the health and the physical activity of Turkish first generation elderly immigrants | Randomised controlled trial | Six cities in the Netherlands  Year: First half of 2001 | 126 people born in Turkey and aged 45 years and over, of whom 92 completed the trial. Welfare services in six Dutch cities | Eight, two hour sessions consisting of health education and exercises. Topics in health education focused on means to maintain a good health. Education was adapted to the culture and knowledge of older Turks and offered by a Turkish peer educator, in Turkish | This article performs the evaluation | Health education and physical exercise improve the mental state of deprived immigrants. Painstaking cultural adaptations to contents and method of delivery are essential to reach this effect |
| **ID 617**  Steptoe et al., 2003 | To measure the effect of brief behavioural counselling in general practice on patients’ consumption of fruit and vegetables in adults from a low income population | Parallel group randomised controlled trial | Primary health centre in a deprived, ethnically  mixed inner city area (UK)  Years 1999-2001 | 271 patients aged 18-70 years without serious illness | Brief individual behavioural counselling based on the stage of change model; time matched nutrition education counselling | This article performs the evaluation | Brief individual counselling in primary care can elicit sustained increases in consumption of fruit and vegetables in low income adults in the general population |
| **ID 656**  Forbes, 2000 | To evaluate a community-based nursing iniative in a deprived inner-city community | Evaluation an intervention with four simple questions | A deprived inner-city community with abundant inequalities (UK) | All the community | The health shop operated for a 14-month period in the main hall of the community centre. A small office was used for private consultations and examinations. Each consultation involved a basic health check and the opportunity to talk through any specific health issues or concerns | This article performs the evaluation | Community nurses can play an important role in supporting people to access health care more effectively |
| **ID 677**  Sykes and Marks,2001 | To test the efficacy of a self-help Cognitive Behaviour Therapy (CBT) for smokers from a deprived area of London | Randomized controlled trial | A deprived area of North London (UK) | 260 adult smokers | “Quit for life” programme: an eclectic combination of 30 CBT and other relevant methods in a self-help package consisting of a handbook, reduction cards, a progress chart and other necessary materials | This article performs the evaluation | This self-help CBT intervention has the potential to reduce the prevalence of smoking among lower socio-economic status smokers |
| **ID 686**  Kennedy, 2001 | To describe the SUPER project (European Food and Shopping Research Project) and its implementation in Liverpool | Description of the design and implementation | Liverpool (UK)  Years 1989-1997 | Population of Liverpool | Different activities to promote healthy eating. For example: nutrition open days in health clinic, school recipe competition, establishment of a local authority food policy network, etc |  | The present study identifies the strengths and weaknesses, opportunities and threats involved in implementing a health promotion approach at local level |
| **ID 704**  Moudgil et al., 2000 | To reports clinical outcomes and quality of life from  a community based project investigating white European and Indian subcontinent  ethnic groups with asthma | Randomised controlled study | Deprived inner city areas of  Birmingham (UK)  Year: 1996 | White European (W/E) and Indian subcontinent  (ISC) asthmatic patients aged 11-59 years | Intervention used an individual asthma education programme (initial session approximately 40 minutes)  which was reinforced after four and eight months. The control groups only attended at the beginning and end of the study | This article performs the evaluation | Active intervention only improved clinical outcomes in the W/E group. Asthma Quality of Life Questionary (AQLQ) scores, although lower in the ISC group, were improved by active intervention in both ethnic groups |
| **ID 740**  Childs et al., 1997 | To assess whether a dietary health education programme could be used within existing health resources to reduce  the incidence of iron deficiency anaemia in an inner city population | Prospective cohort study | Two inner city  areas of west and south Birmingham, both areas of high socioeconomic deprivation (UK) | 1000 children were recruited at birth and randomised into control and intervention groups | Families in the intervention group received specific health education information at key ages by face to face contact using a range of materials. The control group received standard health education | This article performs the evaluation | Anaemia remains a common problem in inner city Birmingham. The dietary health intervention methods used were ineffective in reducing its prevalence |

## Table S2: HEALTHY SETTINGS INTERVENTIONS

| **AUTHOR(S)/**  **YEAR** | **GOAL PAPER** | **STUDY DESIGN OF THE PAPER** | **CITY**  **(COUNTRY) and YEAR** | **TARGET POPULATION OF THE INTERVENTION** | **ACTION/**  **INTERVENTION** | **EVALUATION OF THE INTERVENTION** | **RESULTS / HEALTH OUTCOMES** |
| --- | --- | --- | --- | --- | --- | --- | --- |
| **ID 58**  Lock et al., 2010 | To explore social and behavioural impacts of  English smoke-free legislation (SFL) in different ethnic groups | Qualitative panel study of smokers using in-depth interviews conducted before and after introduction of SFL | North London (UK)    Years 2007-2008 | Turkish, Somali and White communities | Smoke free legislation | Department of Health, 2008  Semple S, 2008 | SFL had positive impacts. Half smoked less and three quitting with no apparent differences in smoking and quitting behaviours between groups; but, notable differences in the social impacts of SFL |
| ID 295 Kimberlee,  2008 | To improve road safety and quality of life in an area of multiple deprivation | Descriptive study.  The target population conducted environmental audits, interactive road safety awareness and citizenship training, and engaged as decision-makers | An area of multiple deprivation in Birmingham (UK)  Year 2006 | 405 students aged 9–11 years from 13 classes  in four primary schools of an area of multiple deprivation in Birmingham (UK) | The project adopted a partnership-based approach involving  school travel plan officers, road safety officers, teachers, dinner ladies, teaching assistants, parents and engineers participation . Activities which were designed to increase road safety and environmental awareness | This article performs the evaluation | To engage young people in decision-making. It has created local space to empower young people to express their views to adults in their community. But the final outcome of this process is yet to be revealed |
| **ID 323**  Gatenby, 2007 | To provide comparative data  regarding the nutritional composition of food served and food eaten in two similar primary schools | A quasi-experimental design. 64 children aged 9–10 years participated from 2 primary schools. Fieldwork was undertaken over 5 days in each school. 320 meals were weighted and photographed before and after consumption  to assess actual intake | Hull (UK)  Year 2004 | All children attending primary and special schools | To provide free healthy  school meals for primary and special schools pupils (approximately 20,500 children) | This article performs the evaluation | Although Hull’s school meals aimed to provide children with adequate nutritional intake, findings demonstrate that, on average, children are served and consume inadequate levels of many nutrients |
| **ID 358**  Ridgers et al., 2007. | To investigate the impact of a playground redesign intervention across time on children's recess physical  activity levels | Quasiexperimental  Intervention evaluation | Large city in England (UK)  Years: 2003-2005 | Fifteen schools in intervention and  eleven schools as matched  socioeconomic controls | To redesign the playground environment based on a multi-coloured zonal design  School playground | This article performs the evaluation | Increase of vigorous and moderate vigorous Physical activity at 6 months |
| **ID 367**  Sagheri et al., 2007 | To compare dental caries levels of schoolchildren stratified in different social classes whose domestic water supply had been fluoridated since birth (Dublin) with those living in an area where fluoridated salt was available (Freiburg) | A representative, random sample of twelve-year-old children was examined and dental caries was recorded using World Health Organization criteria | Dublin (Ireland)  and Freiburg (Germany)  Year 2002 | A total of 699 twelve-year-old children were examined, 377 were children  in Dublin and 322 in Freiburg | Fluoridated drinking water (in Dublin) vs fluoridated salt (in Freiburg) | This article performs the evaluation | The evidence from this study confirmed that water fluoridation  has reduced the gap in dental caries experience between medium and lower social classes in Dublin compared with the greater difference in caries experience between the equivalent social classes in Freiburg. The results from this study established the important role of salt fluoridation where water fluoridation is not feasible |
| **ID 385**  Ridgers et al., 2007 | To investigate the impact of a playground redesign intervention across time on children's recess physical activity levels using combined physical activity measures and to evaluate the potential influence of covariates on the intervention effect | Quasiexperimental intervention evaluation | One large city in the North West England, one of the most deprived areas in the country (UK)  Years 2003-2005 | 232 boys and 238 girls recruited from 26 elementary  schools. Eleven of these schools served as matched  socioeconomic controls | Fifteen schools (130 boys, 126 girls) each received £20,000 to redesign the  playground environment based on the sporting playground zone design. This involved dividing the playground into three specific colour-coded areas: (a) a red sports area, (b) a blue multi-activity area and (c) a yellow quiet play zone | This article performs the evaluation | The results suggest that a playground redesign, which utilizes multi-colour playground markings and physical structures, is a suitable stimulus for increasing children's school recess physical activity levels |
| **ID 709**  DiGuiseppi et al., 1999 | To reduce fires and fire related injuries by increasing the prevalence of functioning smoke alarms in high risk  households | Randomised controlled trial | Camden and Islington, two inner London boroughs (UK)  Years 1997-1998 | Low income and rental households and households with elderly persons or young children | Free smoke alarms and fire safety information were  distributed in intervention wards by community  groups and workers as part of routine activities and by paid workers who visited target neighbourhoods | This article performs the evaluation | A community giveaway programme was successful in distributing 20 050 alarms to high risk households. The programme required a substantial investment of resources. The effectiveness of the programme in reducing fire related injuries is currently being assessed |
| **ID SA20**  Wrigley et al., 2002 | To describe and provide preliminary results from the . first-ever UK study of a major retail provision  on diet in a ‘food desert’ | A ‘before/after’ study of food consumption patterns | Highly deprived area of Seacroft, Leeds (UK)  Year: 2001 | Residents in Seacroft | Improved physical  access to high-quality ‘healthy’ foods for  many of its residents that would follow the  intervention | This article performs the evaluation | A retail provision intervention may have a marked effect on improving the diet of the most ‘at risk’ groups in nutritional terms, and suggests some intriguing mechanisms by which that impact might be translated through into food purchasing/consumption |
| **ID R5**  Somerville et al., 2000 | To evaluate the use of NHS money to improve health by improving housing conditions | A pilot study was assessing health outcomes before and after improving housing conditions | Cornwall (UK) | 72 children with previously diagnosed asthma living in 59 damp houses in Cornwall | The intervention was the installation of central heating. This improved the energy efficiency of the housing. And measures to reduce damp and mould growth within the house | This article performs the evaluation | All respiratory symptoms were significantly reduced after intervention; the greatest reduction was seen in nocturnal cough |
| **ID R6**  Daly et al., 1996 | To compare the haematological and dietary  effects of a follow-on formula with pasteurized cows' milk, in a group of inner city toddlers whose mothers had already switched to pasteurized cows' milk by 6 months of age. | A longitudinal randomised study comparing the haematological and dietary effects of a follow-on formula with pasteurized cows' milk. | Birmingham (UK) | 100 infants who were already receiving  pasteurised cows' milk by 6 months of age deprived inner city area | Infants who were already receiving pasteurised cows' milk by 6 months of age were enrolled and randomised either to receive a follow-on formula or to continue on cows' milk from 6 months until 18 months. At 18 months of age the follow-on formula group returned to cows' milk and both groups were followed up until 24 months | This article performs the evaluation | Infants and toddlers at high risk of iron deficiency are therefore unlikely to become anaemic  if receiving a follow-on formula, although the relative merits of follow-on formula compared with an ordinary infant formula remain uncertain |

## TABLE S3: SOCIOECONOMIC CONTEXT INTERVENTIONS

| **AUTHOR(S)/**  **YEAR** | **GOAL PAPER** | **STUDY DESIGN OF THE PAPER** | **CITY**  **(COUNTRY) and YEAR** | **TARGET POPULATION OF THE INTERVENTION** | **ACTION/**  **INTERVENTION** | **EVALUATION OF THE INTERVENTION** | **RESULTS / HEALTH OUTCOMES** |
| --- | --- | --- | --- | --- | --- | --- | --- |
| ID 345 Daban et al., 2007 | To evaluate primary care reform (PCR) in Barcelona during the year 2000  using 3 preventive practices: anti-smoking advice, blood pressure measurement, and flu vaccination | Cross-sectional, descriptive, observational study | Barcelona (Spain)  Year 2000 | Population of Barcelona | The primary care reform (PCR) favored the primary health care include the health promotion and disease prevention. And to make a health service continuous and integral to the individual, family and community | This article performs the evaluation | PCR is a factor associated with  carrying out preventive practices. No significant disparities between social class or gender were found for those who received the preventive practices |
| **ID 457**  Mujica et al., 2006 | To quantify the cost-effectiveness of day care provision, when compared  with alternative local services used by parents  in achieving the primary outcome of the trial: an increased rate of employment and educational  enrolment in mothers (at 18 months following the offer of a place at the centre) | Economic evaluation conducted alongside  a randomized controlled trial. Participants randomized to receive either high quality day care or to other child care.  Cost of education,  care and productivity gains compared with the effectiveness of the intervention, increased labour force participant in mothers | Borough of Hackney (deprived area) of London (UK)  Years 1999-2000 | 32 children and their families | Early Years Centers provide high quality day care to children aged 6 months to 3.5 years from  socially disadvantaged families | Wiggins, 2003  Toroyan, 2003, 2004 | The value of employment outweighs the costs of health and social services used. However, there is a net cost to the public sector of providing the intervention. Area wide traffic calming is associated with absolute reductions in child pedestrian injury rates and reductions in relative inequalities in child pedestrian injury rates. |
| **ID 573**  Mindell et al. 2004 | To increase the positive and mitigate the negative health impacts of the mayor’s draft transport strategy for London through an HIA | A rapid prospective health impact assessment (HIA) of the penultimate draft of the strategy | London (UK)  Years 1999-2001 | Inhabitants of London | Recommendations from the rapid HIA were fed back into the drafting process | This article performs the evaluation | Almost all the recommendations from the HIA were incorporated into the final strategy: sustainable travel plans for workplaces and schools; priority to infrastructure and services that benefit London’s deprived communities; increased emphasis on promoting walking and cycling and reducing reliance on private cars |
| **ID 608**  Gorman et al., 2003 | This Health impact assessment (HIA) of Edinburgh’s transport policy demonstrates how an HIA can examine how different transport policies can affect different population groupings to varying degrees | This HIA was prospective and can be described as a rapid assessment. It was important that the HIA provided prompt answers and the methods reflect this | Edinburgh (UK) | In this HIA, we only considered impacts borne by the resident population of the City of Edinburgh Council (CEC) area | An expert group was formed that consisted  of council transport planners, health board and university public health staff. Two group members did the background work including literature review and policy analysis. The main categories of health  impact to be explored in this HIA were identified  from the literature review and analysis of the  transport policy. The whole group then met for two half days to conduct the HIA | In our work, we did not further quantify the health impacts by, for example, estimating the  number of people who would bear each impact.  The information we gathered was enough to make the key health impacts explicit and describe their distribution in the population | The HIA suggested that greater spend on public transport and supporting sustainable modes of transport was beneficial to health, and offered scope to reduce inequalities.  This message was understood by the City Council and influenced the development of the city’s transport and land-use strategies.  A major conclusion of the HIA is that having a contemporary transportation policy which reduces private car use by encouraging cycling, walking and  public transport is beneficial to all, but particularly to the most disadvantaged groups |
| **ID R3**  Toroyan et al., 2003 | To assess the effects of providing day-care facilities for young children on the health and welfare of disadvantaged families | Randomised controlled trial. Eligible children  from the application list to a day-care facility were randomly allocated to receive a day-care place or not | Early Years day-care centre in Borough of  Hackney, London (UK) | 120 mothers and 143 eligible children  (aged between 6 months and 3.5 years) | A place at the centre, which provided high quality day care. Control families used other child care that they secured for themselves | This article performs the evaluation | The provision of child day-care may have increased maternal employment, but it did not seem  to increase household income. The results suggest that providing day-care may be insufficient as a strategy to reduce poverty |
| **ID R4**  Linares, 2001 | To describe food services and insertion plan for the homeless carried out by the Caritas Programme for  the Homeless In Spain | Descriptive | Different cities in Spain | Homeless population | In Spain 315 centres offer meals for 20 000 people, most of them along with other services such as reception, residential premises or day-care centres. Caritas is responsible for 42% of them | No | Overall, it is easier to get a lunch than any other meal during the day, followed by dinner, while it is difficult to get a breakfast. Social dining rooms are valuable places for a first contact (first stage of the insertion process) and even to start the second stage (personal recovery) |

## Table S4: PHYSICAL CONTEXT INTERVENTIONS

| **AUTHOR(S)/**  **YEAR** | **GOAL PAPER** | **STUDY DESIGN OF THE PAPER** | **CITY**  **(COUNTRY) and YEAR** | **TARGET POPULATION OF THE INTERVENTION** | **ACTION/**  **INTERVENTION** | **EVALUATION OF THE INTERVENTION** | **RESULTS / HEALTH OUTCOMES** |
| --- | --- | --- | --- | --- | --- | --- | --- |
| **ID 478**  Thomson et al., 2006 | To synthesise data on the impact on health and key socioeconomic determinants of health and health inequalities reported in evaluations of national UK regeneration programmes | Review. Synthesis of evidence | National urban regeneration program areas  Search 1980-2004 | Urban regeneration program areas  inhabitants | Urban regeneration programs: renewal, housing, employment, training. | This article synthetises evaluation | Little evidence of the impact of national urban regeneration investment on socioeconomic or health outcomes.  Where impacts have been assessed, these are often small and positive but adverse impacts have also occurred |
| **ID 514**  Jones et al., 2005 | To determine whether area wide traffic calming distribution reflects known inequalities in child  pedestrian injury rates. To determine whether traffic calming is associated with changes in childhood  pedestrian injury rates | Small area ecological study, longitudinal analysis of injury rates with cross sectional analysis of  traffic calming and method of travel to school | Two cities in the United Kingdom.  Years 1992 -2000 | 4–16 year old children | Traffic calming (speed humps, road narrowings, road closures, and  speed cushions) | This article performs the evaluation | Injury rates among the most  deprived dropped from 9.42 to 5.07 from 1992–94 to 1998–2000 (95% CI for change 2.82 to 5.91). In city B, the traffic calming ratio of the most to least deprived fourth was 1.88 (95% CI 1.46 to 2.42); injury rates in the deprived areas dropped from 8.92 to 7.46 (95% CI for change 20.84 to 3.77) |
| **ID 679**  Thomson et al., 2001 | To review the evidence on the effects of interventions to improve housing on health | Systematic review of experimental and  non­experimental housing intervention studies that  measured quantitative health outcomes | UK  Year: Studies dating from 1887 | 18 studies were reviewed that studied the health effects of housing  improvements | The interventions included rehousing, refurbishment, and energy efficiency measures | Not applicable | Many studies showed health gains after the intervention, but the small study populations and lack of controlling for confounders limit the generalisability of these findings |
| **ID R2**  Blackman et al., 2001 | To assess the effects of neighbourhood renewal on residents’ health | A before-and-after study of the effects of neighbourhood renewal on residents’ health | Neighbourhood Renewal Area (NRA) in the west end of  Newcastle Upon Tyne (UK)  Years: 1992-1997 | People who live in house deprivation conditions | Environmental and security improvements funded  by the renewal programme | This article performs the evaluation | Following the renewal work, improvements occurred in both adults’ and children’s mental health, and smoking  declined sharply. Respiratory health did not improve and there was no change in use of health services |

## Table S5: COMBINED APPROACH INTERVENTIONS

| **AUTHOR(S)/**  **YEAR** | **GOAL PAPER** | **STUDY DESIGN OF THE PAPER** | **CITY**  **(COUNTRY) and YEAR** | **TARGET POPULATION OF THE INTERVENTION** | **ACTION/**  **INTERVENTION** | **EVALUATION OF THE INTERVENTION** | **RESULTS / HEALTH OUTCOMES** |
| --- | --- | --- | --- | --- | --- | --- | --- |
| **ID 112**  Ritsatakis, 2009 | To examine how cities included in Healthy Cities translated the principle of equity in health into action | Using information designed to help evaluate Phase III (1998–2002) of the WHO European Healthy Cities Network, plus documentation from city programmes and websites, an attempt is made to assess how far stakeholders in cities understood the concept of equity in health, had the political will to tackle the issue and the types of action undertaken | Forty-one cities of Europe  Year 2002 | Mainly on  support for vulnerable groups | The majority were lifestyle oriented methods or those to improve access to care | There was little experience  of evaluating the impact of interventions | Cities focused mainly on support for vulnerable groups, and a wide range of actions were being implemented, including lifestyle oriented methods or those to improve access to care. Few cities made the necessary shift towards more upstream policies to tackle determinants of health such as poverty, unemployment and housing |
| **ID 123**  Muckelbauer et al., 2009  Obesity facts | To analyze the effect of a combined environmental and educational intervention solely promoting water consumption on the Incidence of overweight  among school children | A randomized, controlled cluster trial with 1 intervention arm and 1 control arm considered schools as cluster units of intervention | In deprived  neighbourhood of Dortmund and Essen (Germany)  Years 2006-2007 | Children attending the  second and third grades of elementary schools | In intervention schools (N = 17), water fountains were installed, each child received a water bottle, and teachers performed classroom lessons to promote water consumption. Control schools (N = 15) did not receive any intervention | This article performs the evaluation | A simple dietary intervention with the sole focus on the promotion of drinking water effectively reduced the incidence of overweight among school children |
| **ID 195**  Muckelbauer et al., 2009 | To test whether a combined environmental and educational intervention solely promoting water consumption was effective in preventing overweight among children in elementary school | A randomized, controlled cluster trial with 1 intervention arm and 1 control arm considered schools as cluster units of intervention | In deprived  neighbourhoods of Dortmund and Essen (Germany)  Years 2006-2007 | Children attending the  second and third grades of elementary schools | Water fountains were installed and teachers presented 4 prepared classroom lessons | This article performs the evaluation | After the intervention, the risk of overweight was reduced by 31% in the intervention group, compared with the control group |
| **ID 330**  Bowen,  2007 | To ensure that strategy proposals endeavoured  to reduce health inequalities, mitigate negative health outcomes and increase positive health outcomes | A workshop and literature review were carried out to carry out rapid HIA as a part of the policy-making process | London (UK)  Years 2001-2003 | Population of London | The London Health Commission undertook health impact assessments (HIAs) on each of the draft strategies and on several other non-statutory draft strategies the Mayor developed between 2001 and 2003 | The London Health Commission commissioned an evaluation of the HIAs undertaken, which found that the use of HIA did have an impact on incorporating health considerations  into the strategies | Changes were made to the  strategies as a result of recommendations |
| **ID 551**  Wiggins et al., 2004 | To determine whether increased postnatal  support could influence maternal and child health  outcomes | A randomised controlled trial comparing maternal and child health outcomes for women offered either of the support interventions with those for control women receiving standard services only | Deprived enumeration districts in  the London boroughs of Camden and Islington (UK)  Year 1999 | Women were eligible for the trial if they gave birth between 1 January and 30 September 1999 | A programme of visits from health visitors trained in supportive listening [Support Health Visitor (SHV)] and the services of local community support organisations [Community  Group Support (CGS)] | This article performs the evaluation | There was no evidence of impact on the primary outcomes of either intervention. The support health visitor intervention was popular with women, and was associated with improvement in some of the secondary outcomes |
| **ID 561**  Diez and Peiró, 2004 | Three interventions to reduce health inequalities in Spain are described:  A. Investment Plan in Puente and Villa of Vallecas  B. Programme to promote health in ethnic minorities in Navarra  C. Olimpia project to prevent HIV/AIDS and other communicable diseases in women who perform prostitution in the Autonomous Community of Galicia | To balance an area with social deprivation  A person is selected from each basic health area to intervene in the education of a community according to the objectives established in each zone  A study of the local conditions of prostitution to bring social and health care services closer | Vallecas, Madrid (Spain). Years:2000-2005  Navarra (Spain)  Years: from 1988 onward  Vigo, Pontevedra, Orense, Lugo y Santiago  de Compostela (Spain)  Years: 1997 onward | The areas of Vallecas with social deprivation  Population of gipsy ethnic background from Spain and Portugal in Navarra  Women who perform prostitution in Galicia | An investment of 18.000 million Pesetas were used in different areas of the of the social and economic structures in the zone  A peer and health agents education programme  A mobile unit carried out the intervention and women who request bloodwork were followed up by the public health and social network. Workshops were also performed in 2007 | The evaluation has not been carried out  There has not been an evaluation carried out with health indicators  None | Unknown  There are no indicators but results are considered as satisfying  677 women have received care 1.431 times |
| **ID 649**  Gray, 2002 | To evaluate the Family Support Services (FSSs) | Ethnographic investigation | Tower Hamlets, a multi-racial  area in east London (UK) | Population of Tower Hamlets | FSSs includes three projects: Family Support,  Building Bridges and Quality Protects. The service expects to work with some 10-13 families  at any one time for 3-6 months | This article is a qualitative assessment | The evaluation demonstrates that FSSs provided a highly effective service and gave very good quality support to families. Families indicated that FSSs made a variety of substantial positive outcomes in  their health and social care. Professionals said FSSs  contributed to effective service delivery, co-ordination and community support |
| **ID 761**  Díez et al., 1995 | Evaluation of the population impact of a social and health maternal and child intervention | A quasi-experimental design with a nonequivalent control group and multiple measurements was used | A low income urban area (Ciutat Vella) in Barcelona (Spain)  Years 1987-1992 | Pregnant women and children living in Ciutat Vella | Intervention was based on increasing access to health and social services. It is based on early detection and monitoring of pregnant women and newborns through home visits and referrals to social and health services. | This article performs the evaluation | The significant differences found previously to the program between the mortality rates (infant and perinatal) between Ciutat Vella and the rest of the city disappeared with the development of the program |
| ID SA1  Abbas and Anwar, 2005 | To explore the effectiveness of race equality policies | Secondary analysis of documentary  evidence and interviews with key actors | Birmingham, UK | Ethnic minorities living in Birmingham | Council legislation against race inequalities since 1972 | This article performs the evaluation | Ethnic minorities are disadvantaged in education, the labour market, and in relation to health and housing.  Race equality policies remain ineffective and a great deal more is required |
| **ID R1**  Diez et al, 1996 | To evaluate a social care and health follow-up programme targeting homeless tuberculosis patients in Ciutat Vella District, Barcelona, which covered 210 patients from 1987 to 1992 | The differential tuberculosis incidence rate between Ciutat Vella and the other districts of Barcelona, the percentage of successfully completed treatments and the days of hospitalization saved by the programme  were measured | A low income urban area (Ciutat Vella) in Barcelona (Spain)  Years: 1987-1992 | Homeless tuberculosis patients | Directly observed treatment, primary health care and, if necessary, accommodation was provided | This article performs the evaluation | There was a significant decrease in the tuberculosis incidence rate among homeless patients in Ciutat  Vella. The programme appears to be both effective and efficient, as it has enabled a large number of  homeless patients to complete their treatment successfully, at the same time saving twice the amount of funds  invested |

**54 papers selected**

(1) Abbas T, Anwar M (2005). An Analysis of Race Equality Policy and Practice in the City of Birmingham, UK Local Government Studies 31(1):53-68.

(2) Bellary S, O'Hare JP, Raymond NT, Gumber A, Mughal S, Szczepura A, et al. (2008). Enhanced diabetes care to patients of south Asian ethnic origin (the United Kingdom Asian Diabetes Study): a cluster randomised controlled trial. Lancet 371(9626):1769-1776.

(3) Blackman T, Harvey J, Lawrence M, Simon A (2001). Neighbourhood renewal and health: evidence from a local case study. Health Place 7(2):93-103.

(4) Bowen C (2007). Health impact assessments in London: assessing the London Mayoral strategies. N S W Public Health Bull 18(9-10):185-187.

(5) Childs F, Aukett A, Darbyshire P, Ilett S, Livera LN (1997). Dietary education and iron deficiency anaemia in the inner city. Arch Dis Child 76(2):144-147.

(6) Cochrane T, Davey RC (2008). Increasing uptake of physical activity: a social ecological approach. J R Soc Promot Health 128(1):31-40.

(7) Daban F, Pasarin MI, Rodriguez-Sanz M, Garcia-Altes A, Villalbi JR, Cano-Serral G, et al. (2007). Evaluation of the primary health care reform: preventive practices and inequalities. Aten Primaria 39(7):339-346.

(8) Daly A, MacDonald A, Aukett A, Williams J, Wolf A, Davidson J, et al. (1996). Prevention of anaemia in inner city toddlers by an iron supplemented cows' milk formula. Arch Dis Child 75(1):9-16.

(9) Davies GM, Duxbury JT, Boothman NJ, Davies RM (2007). Challenges associated with the evaluation of a dental health promotion programme in a deprived urban area. Community Dent Health 24(2):117-121.

(10) Diez E, Claveria J, Serra T, Cayla JA, Jansa JM, Pedro R, et al.(1996). Evaluation of a social health intervention among homeless tuberculosis patients Tuber Lung Dis 77(5):420-424.

(11) Diez E, Peiro R (2004). Interventions to reduce health inequalities. Gac Sanit 18 (Suppl 1):158-167.

(12) Diez E, Villalbi JR, Benaque A, Nebot M (1995). Inequalities in maternal-child health: impact of an intervention. Gac Sanit 9(49):224-231.

(13) DiGuiseppi C, Slater S, Roberts I, Adams L, Sculpher M, Wade A, et al. (1999). The "Let's Get Alarmed!" initiative: a smoke alarm giveaway programme. Inj Prev 5(3):177-182.

(14) El Fakiri F, Bruijnzeels MA, Uitewaal PJ, Frenken RA, Berg M, Hoes AW (2008). Intensified preventive care to reduce cardiovascular risk in healthcare centres located in deprived neighbourhoods: a randomized controlled trial. Eur J Cardiovasc Prev Rehabil 15(4):488-493.

(15) Forbes A. (2000). A community nurse-led project to tackle health inequalities. Br J Community Nurs. 5(12):610-8.

(16) Gatenby LA (2007). Nutritional content of school meals in Hull and the East Riding of Yorkshire: a comparison of two schools. J Hum Nutr Diet 20(6):538-548.

(17) Gorman D, Douglas MJ, Conway L, Noble P, Hanlon P (2003). Transport policy and health inequalities: a health impact assessment of Edinburgh's transport policy. Public Health 117(1):15-24.

(18) Gray B (2002). Working with families in Tower Hamlets: an evaluation of the Family Welfare Association's Family Support Services. Health Soc Care Community 10(2):112-122.

(19) Greenhalgh T, Collard A, Begum N (2005). Sharing stories: complex intervention for diabetes education in minority ethnic groups who do not speak English. BMJ 330(7492):628.

(20) Griffiths C, Foster G, Barnes N, Eldridge S, Tate H, Begum S, et al (2004). Specialist nurse intervention to reduce unscheduled asthma care in a deprived multiethnic area: the east London randomised controlled trial for high risk asthma (ELECTRA). BMJ 328(7432):144.

(21) Hajek P, Humphrey K, McRobbie H (2010). Using group support to complement a task-based weight management programme in multi-ethnic localities of high deprivation. Patient Educ Couns 80(1):135-137.

(22) Harting J, Kunst AE, Kwan A, Stronks K (2011). A 'health broker' role as a catalyst of change to promote health: an experiment in deprived Dutch neighbourhoods. Health Promot Int 26(1):65-81.

(23) Horgan JM, Blenkinsopp A, McManus RJ (2010). Evaluation of a cardiovascular disease opportunistic risk assessment pilot ('Heart MOT' service) in community pharmacies. J Public Health (Oxf) 32(1):110-116.

(24) Jackson RJ, Newman HN, Smart GJ, Stokes E, Hogan JI, Brown C, et al (2005). The effects of a supervised toothbrushing programme on the caries increment of primary school children, initially aged 5-6 years. Caries Res 39(2):108-115.

(25) Jones SJ, Lyons RA, John A, Palmer SR (2005). Traffic calming policy can reduce inequalities in child pedestrian injuries: database study. Inj Prev 11(3):152-156.

(26) Kennedy LA (2001). Community involvement at what cost? local appraisal of a pan-European nutrition promotion programme in low-income neighbourhoods. Health Promot Int 16(1):35-45.

(27) Kerr C, Murray E, Noble L, Morris R, Bottomley C, Stevenson F, et al (2010). The potential of Web-based interventions for heart disease self-management: a mixed methods investigation. J Med Internet Res 12(4):e56.

(28) Kimberlee R (2008). Streets ahead on safety: young people's participation in decision-making to address the European road injury 'epidemic'. Health Soc Care Community 16(3):322-328.

(29) Linares E (2001). Food services for the homeless in Spain: Caritas Programme for the Homeless.Public Health Nutr 4(6A): 1367-9

(30) Lock K, Adams E, Pilkington P, Duckett K, Gilmore A, Marston C. (2010). Evaluating social and behavioural impacts of English smoke-free legislation in different ethnic and age groups: implications for reducing smoking-related health inequalities. Tob Control 19(5):391-397.

(31) McDonald C, Lambert J, Nayagam D, Welz T, Poulton M, Aleksin D, et al (2007). Why are children still being infected with HIV? Experiences in the prevention of mother-to-child transmission of HIV in south London. Sex Transm Infect 83(1):59-63.

(32) McIntosh J, Shute J (2007). The process of health visiting and its contribution to parental support in the Starting Well demonstration project. Health Soc Care Community 15(1):77-85.

(33) Mindell J, Sheridan L, Joffe M, Samson-Barry H, Atkinson S (2004). Health impact assessment as an agent of policy change: improving the health impacts of the mayor of London's draft transport strategy. J Epidemiol Community Health 58(3):169-174.

(34) Mohiddin A, Cawley L, Chow Y, Wallis R (2006). Life as a league table bottom dweller: teenage pregnancy in Lambeth. J Public Health (Oxf) 28(4):304-308.

(35) Moudgil H, Marshall T, Honeybourne D (2000). Asthma education and quality of life in the community: a randomised controlled study to evaluate the impact on white European and Indian subcontinent ethnic groups from socioeconomically deprived areas in Birmingham, UK. Thorax 55(3):177-183.

(36) Muckelbauer R, Libuda L, Clausen K, Reinehr T, Kersting M (2009). A simple dietary intervention in the school setting decreased incidence of overweight in children. Obes Facts 2(5):282-285.

(37) Muckelbauer R, Libuda L, Clausen K, Toschke AM, Reinehr T, Kersting M (2009). Promotion and provision of drinking water in schools for overweight prevention: randomized, controlled cluster trial. Pediatrics 123(4):e661-7.

(38) Mujica Mota R, Lorgelly PK, Mugford M, Toroyan T, Oakley A, Laing G, et al (2006). Out-of-home day care for families living in a disadvantaged area of London: economic evaluation alongside a RCT. Child Care Health Dev 32(3):287-302.

(39) Rae M (2006). Health inequalities--a sustainable development issue. Public Health 120(12):1106-1109.

(40) Reijneveld SA, Westhoff MH, Hopman-Rock M (2003). Promotion of health and physical activity improves the mental health of elderly immigrants: results of a group randomised controlled trial among Turkish immigrants in the Netherlands aged 45 and over. J Epidemiol Community Health 57(6):405-411.

(41) Ridgers ND, Stratton G, Fairclough SJ, Twisk JW (2007). Children's physical activity levels during school recess: a quasi-experimental intervention study. Int J Behav Nutr Phys Act 4:19.

(42) Ridgers ND, Stratton G, Fairclough SJ, Twisk JW (2007). Long-term effects of a playground markings and physical structures on children's recess physical activity levels. Prev Med 44(5):393-397.

(43) Ritsatakis A (2009). Equity and social determinants of health at a city level. Health Promot Int 24 (Suppl 1):i81-i90.

(44) Sagheri D, McLoughlin J, Clarkson JJ (2007). A comparison of dental caries levels in two communities with different oral health prevention strategies stratified in different social classes. J Public Health Dent 67(1):1-7.

(45) Schuring M, Burdorf A, Voorham AJ, der Weduwe K, Mackenbach JP (2009). Effectiveness of a health promotion programme for long-term unemployed subjects with health problems: a randomised controlled trial. J Epidemiol Community Health 63(11):893-899.

(46) Somerville M, Mackenzie I, Owen P, Miles D (2000). Housing and health: does installing heating in their homes improve the health of children with asthma?. Public Health 114(6):434-439.

(47) Steptoe A, Perkins-Porras L, McKay C, Rink E, Hilton S, Cappuccio FP (2003). Behavioural counselling to increase consumption of fruit and vegetables in low income adults: randomised trial. BMJ 326(7394):855.

(48) Sykes CM, Marks DF (2001). Effectiveness of a cognitive behaviour therapy self-help programme for smokers in London, UK. Health Promot Int 16(3):255-260.

(49) Thomson H, Atkinson R, Petticrew M, Kearns A (2006). Do urban regeneration programmes improve public health and reduce health inequalities? A synthesis of the evidence from UK policy and practice (1980-2004). J Epidemiol Community Health 60(2):108-115.

(50) Thomson H, Petticrew M, Morrison D (2001). Health effects of housing improvement: systematic review of intervention studies. BMJ 323(7306):187-190.

(51) Toroyan T, Roberts I, Oakley A, Laing G, Mugford M, Frost C (2003). Effectiveness of out-of-home day care for disadvantaged families: randomised controlled trial. BMJ 327(7420):906.

(52) Tubert-Jeannin S, Lecuyer MM, Manevy R, Pegon-machat E, Decroix B (2008). Evaluation of an oral health promotion program after one year of implementation in a nursery school. Sante Publique 20(1):7-17.

(53) Wiggins M, Oakley A, Roberts I, Turner H, Rajan L, Austerberry H, et al (2004). The Social Support and Family Health Study: a randomised controlled trial and economic evaluation of two alternative forms of postnatal support for mothers living in disadvantaged inner-city areas. Health Technol Assess 8(32):iii, ix-x, 1-120.

(54) Wrigley N, Warm D, Margetts B, Whelan A (2002). Assessing the Impact of Improved Retail Access on Diet in a 'Food Desert': A Preliminary Report. Urban Stud 39(11):2061-2082.
